# A novel deep learning model for glioma epilepsy associated with the identification of human cytomegalovirus infection injuries based on head MR

**DOI:** 10.3389/fmicb.2023.1291692

**Published:** 2023-11-02

**Authors:** Wei Wang, Xuanyi Li, Lou Ye, Jian Yin

**Affiliations:** ^1^Department of Neurosurgery, The Second Affiliated Hospital of Dalian Medical University, Dalian, Liaoning, China; ^2^Department of Radiology, Shengjing Hospital of China Medical University, Shenyang, Liaoning, China; ^3^Department of Hematology, Da Qing Long Nan Hospital, Daqing, Heilongjiang, China; ^4^Epileptic Center of Liaoning, The Second Affiliated Hospital of Dalian Medical University, Dalian, Liaoning, China

**Keywords:** human cytomegalovirus, glioma, epilepsy, deep learning, injury assessment

## Abstract

**Purpose:**

In this study, a deep learning model was established based on head MRI to predict a crucial evaluation parameter in the assessment of injuries resulting from human cytomegalovirus infection: the occurrence of glioma-related epilepsy. The relationship between glioma and epilepsy was investigated, which serves as a significant indicator of labor force impairment.

**Methods:**

This study enrolled 142 glioma patients, including 127 from Shengjing Hospital of China Medical University, and 15 from the Second Affiliated Hospital of Dalian Medical University. T1 and T2 sequence images of patients’ head MRIs were utilized to predict the occurrence of glioma-associated epilepsy. To validate the model’s performance, the results of machine learning and deep learning models were compared. The machine learning model employed manually annotated texture features from tumor regions for modeling. On the other hand, the deep learning model utilized fused data consisting of tumor-containing T1 and T2 sequence images for modeling.

**Results:**

The neural network based on MobileNet_v3 performed the best, achieving an accuracy of 86.96% on the validation set and 75.89% on the test set. The performance of this neural network model significantly surpassed all the machine learning models, both on the validation and test sets.

**Conclusion:**

In this study, we have developed a neural network utilizing head MRI, which can predict the likelihood of glioma-associated epilepsy in untreated glioma patients based on T1 and T2 sequence images. This advancement provides forensic support for the assessment of injuries related to human cytomegalovirus infection.

## 1. Introduction

**Figure 1:**
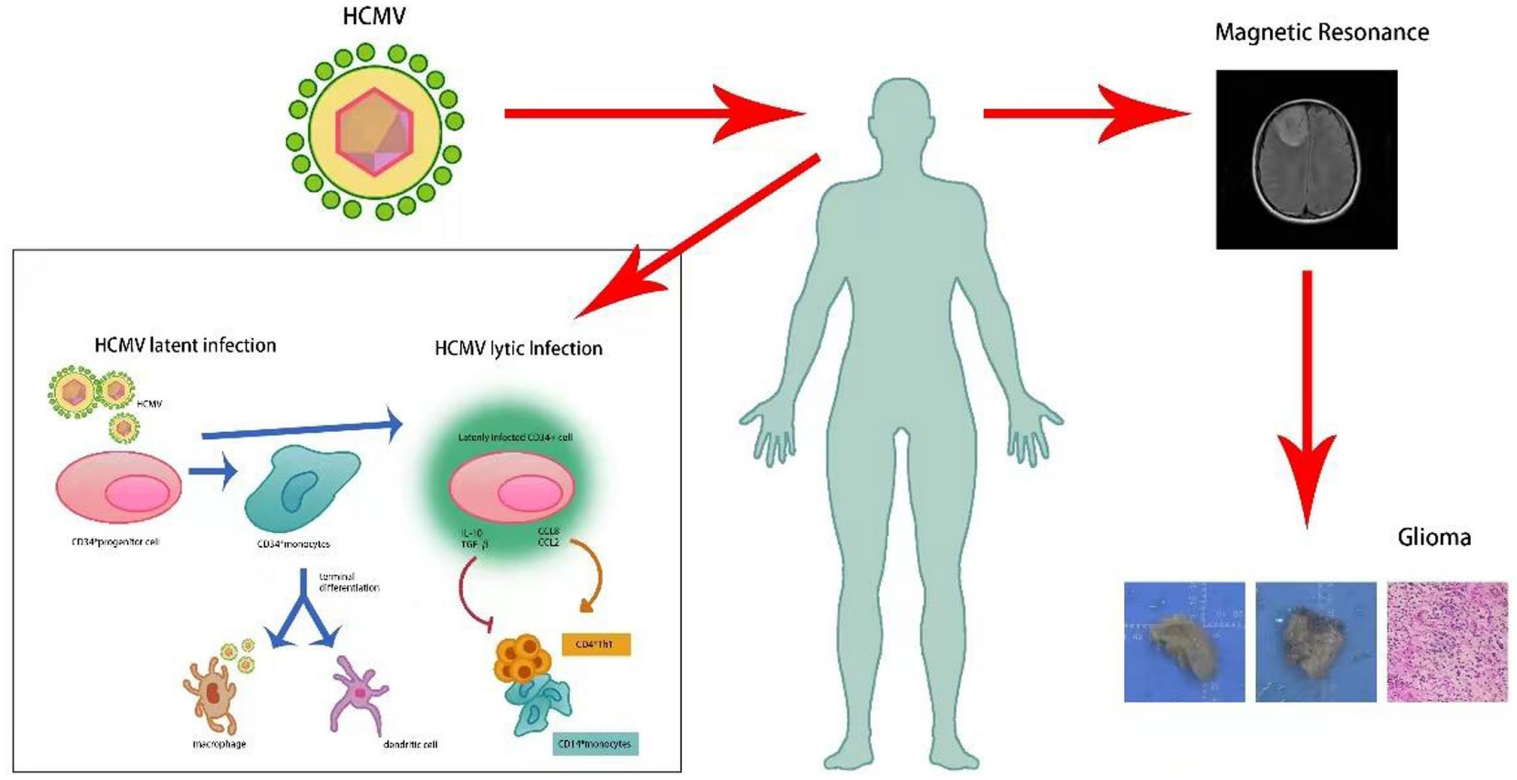
Human cytomegalovirus reinfection is associated with a potential glioma risk.

Human cytomegalovirus (HCMV) is a double-stranded DNA herpesvirus, while HCMV infections typically pose minimal harm to healthy individuals, the immune system of infected individuals can usually eradicate the virus in the early stages of infection, resulting in mild or asymptomatic presentations. However, this virus presents a considerable risk to immunocompromised individuals (Nogalski et al., 2014). The mechanism through which human cytomegalovirus (HCMV) inflicts harm is via viremia, wherein an elevation in viral load surpasses a specific threshold, consequently causing patients to manifest various clinical symptoms (Stagno et al., 1975). Generally, upon initial infection with human cytomegalovirus (HCMV), individuals experience primary infection. Subsequently, the virus enters a latent phase. The capacity to establish lifelong latent infection in the host is a characteristic hallmark of HCMV infection (Goodrum, 2016). Hence, when individuals experience a decline in their immune defenses, clinical symptoms can reappear in patients (Jenks et al., 2021). Re-infection remains a possibility if previously infected individuals come into contact with the virus again. In addition to the harm caused directly by cytomegalovirus-induced viremia, recent research suggests a potential association between human cytomegalovirus and glioma, as shown in Figure 1 (Cobbs, 2011). Glioblastoma, a broad term encompassing neuroepithelial tumors originating from the neural glial or supporting cells of the brain, stands as the most fatal and prevalent primary brain tumor in adults. Among adults, the most common malignant primary glioma is the astrocytic glioblastoma (Louis et al., 2016). Excluding genetic factors, the specific risk factors contributing to the onset of glioblastoma remain unclear. Nevertheless, there is substantial evidence indicating the presence of human cytomegalovirus DNA and mRNA in tumor samples from glioblastoma cases (Lawler, 2015).

Forensic microbiology is a branch of forensic science that involves the examination of various microbial characteristics to infer the specific microbial source and transmission pathway, thereby providing microbiological evidence for legal purposes (Yuan et al., 2023). In forensic practice, forensic biology can trace the origin of a pathogen in infected individuals by identifying the biological samples of both the source and the infected person (Murugesan et al., 2023). As awareness of public health grows, passive and preventable viral infections are gaining increased attention. It is becoming increasingly common for infected individuals to seek financial compensation from transmitters or individuals/collectives responsible for creating susceptible environments (El Helou et al., 2022). In cases involving pathogen-related infections, the accurate forensic assessment of injuries is undoubtedly a focal point of forensic work. However, with the advancement of technology, forensic experts can now employ algorithms based on machine learning and deep learning to identify individual humans and microbial organisms (Yuan et al., 2023). In the context of injury assessment related to glioma caused by cytomegalovirus, particular attention must be given to whether the patient experiences epilepsy. This is crucial because the occurrence of epilepsy in infected individuals significantly affects the forensic evaluation of their injuries. Firstly, epilepsy itself is a critical assessment parameter in injury evaluation (Stewart et al., 2004). In Chinese law, according to current Chinese law, labor capacity assessment is divided into ten levels. Although it is necessary to consider the patient’s medical dependency, functional impairment, and self-care abilities comprehensively, the law specifically stipulates that, depending on the severity of epilepsy, the patient’s loss of labor capacity is categorized as level four, level six, and level nine. Additionally, research has indicated a significant survival benefit for patients with epilepsy diagnosed within 1 year before the diagnosis of glioma compared to patients without a history of epilepsy. This association holds substantial significance for both low-grade gliomas (Grade II) and high-grade gliomas (Grade III and IV; Marku et al., 2022). Hence, concerning injury assessment, the occurrence of epilepsy in glioma patients unquestionably implies a shortened life expectancy for the patient. However, it is not a certainty that all glioma patients will experience epilepsy. Literature suggests that approximately one-quarter to one-half of brain tumors are accompanied by seizures (Pace et al., 1998; Lynam et al., 2007). Currently, the relationship between brain tumors and epileptic seizures remains unclear. The incidence of epileptic seizures depends on the tumor type and grade, to some extent on the patient’s age, and the location of the tumor. Glioblastoma and tumor-related epilepsy share common pathophysiological mechanisms that can drive tumor progression and the onset of seizures (Huberfeld and Vecht, 2016). For clinicians, the primary treatment approach for glioma patients is surgical tumor removal. However, for a substantial number of patients, this approach obscures potential indicators for injury assessment. Therefore, the development of an early, non-invasive method for assessing glioma-related epilepsy is of paramount importance for forensic injury assessment of patients.

Currently, it is considered that for epilepsy patients, an MRI (Magnetic Resonance Imaging) should be conducted at the initial onset and diagnosis of epilepsy, and it should be repeated during subsequent treatment (Bernasconi et al., 2019). As for tumor-related epilepsy, MRI is even more crucial because it can assess tumor type, tumor growth rate, location, and tumor burden, providing valuable information in this context (Chen et al., 2018). MRI plays a pivotal role in the diagnosis and treatment of brain tumors in humans (Fink et al., 2015). MRI can assess the extent of tumor invasion and recurrence by reflecting the edema and capsule region of gliomas (Petrecca et al., 2013). In gliomas, diagnoses based on MR imaging possess a high level of reliability and are widely utilized to identify the location and size of gliomas. This is considered a crucial method for diagnosing and assessing gliomas (Martínez-Garcia et al., 2018). A study has shown that a radiomic model based on diffusion and perfusion-weighted MRI images can predict the isocitrate dehydrogenase (IDH) mutation and tumor invasiveness in diffuse low-grade gliomas (Kim et al., 2020). This indicates that for gliomas, MRI images can comprehensively reflect the medical information related to the tumor. Currently, artificial intelligence based on medical imaging has made significant advancements in various fields, including gliomas. A study utilizing radiological features extracted from T2 sequences of 233 glioma patients was able to predict patient survival (Qian et al., 2018). In a study comprising 212 patients, radiomic features extracted from patient MRI scans were employed to define glioma subtypes based on tumor grade, IDH mutation, and 1p/19q co-deletion (Li et al., 2022). In contrast, deep learning methods do not require the prior selection of features but rather have the capacity to learn which features are most relevant for classification and/or prediction. Concerning the deep learning approach, various medical images can be employed for glioma diagnosis. A study utilized 990,267 histological H&E stained images from 79 patients for glioma classification (Jin et al., 2021). A study employed diffusion tensor imaging to classify the risk of glioma patients (Yan et al., 2021). However, the majority of deep learning research still predominantly utilizes MRI data, and there are now numerous publicly available datasets to support this research direction (Menze et al., 2014).

This study included data from two centers and developed a neural network model that predicts whether patients will develop secondary epilepsy due to glioma by incorporating early brain MR images of glioma patients. This provides forensic injury assessment support without interfering with patient treatment. To validate the model’s effectiveness, it was compared with a machine learning model constructed using tumor images segmented by experts.

## 2. Materials and methods

### 2.1. Data collection

**Figure 2:**
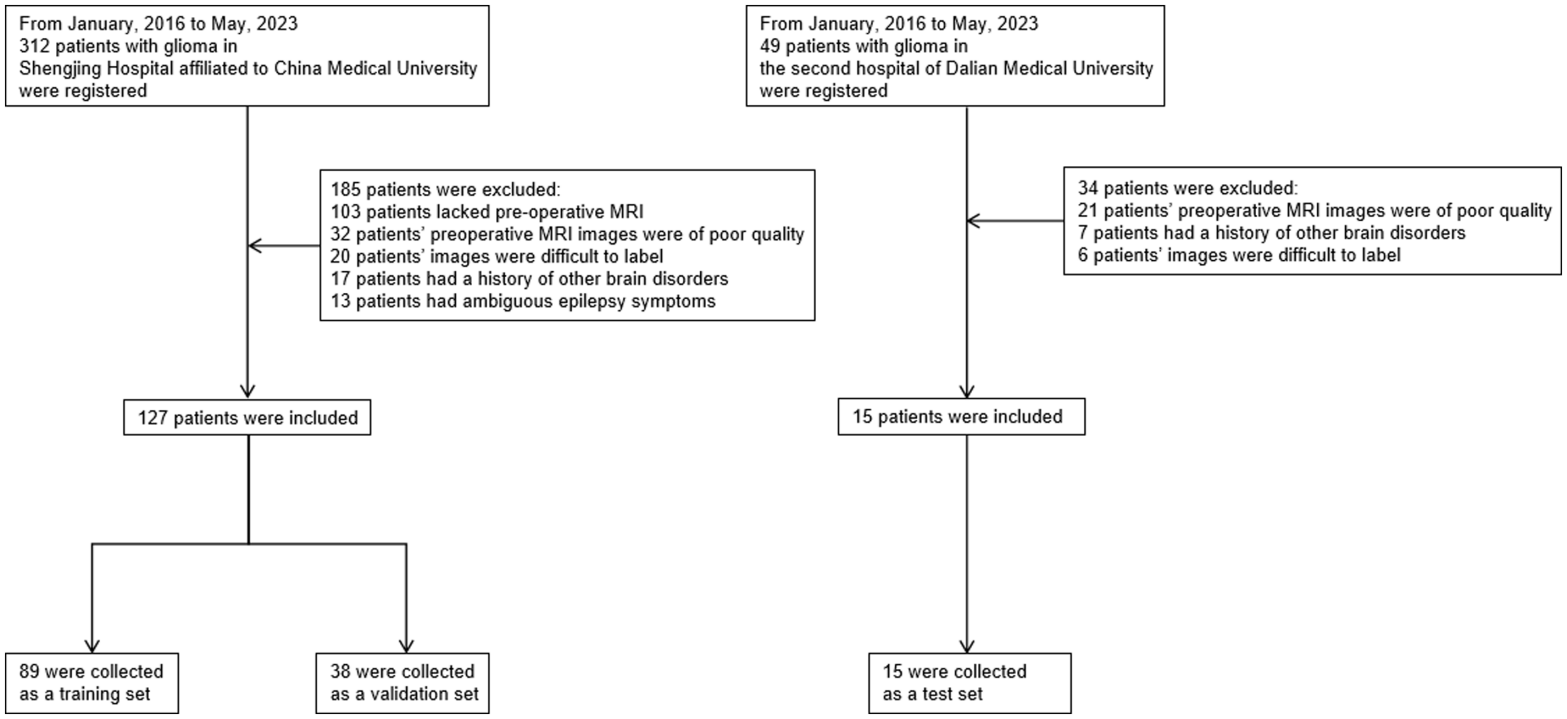
Flowchart of the sample collection process in this study, which involved data from two centers. We primarily excluded patients with poor image quality, those with brain diseases, and patients with low-grade gliomas that were difficult to annotate on MRI. To improve data quality, patients with a history of epilepsy or those whose epilepsy diagnosis was challenging based on clinical symptoms were also excluded.

This study has been approved by the Ethics Committee of China Medical University Shengjing Hospital and the Ethics Committee of Dalian Medical University Second Hospital, with ethics reference numbers 2023PS1002K and 2,023,216, respectively. We conducted a search on patients at Shengjing Hospital of China Medical University from January 2016 to May 2023. Patients were included based on the following criteria: they underwent an MRI within the first week of hospitalization and subsequently underwent surgery or biopsy, leading to a pathological diagnosis of primary glioma. Clinical symptoms of epilepsy were assessed by clinicians upon the patient’s initial hospitalization. The diagnosis of epilepsy in patients was primarily based on their clinical presentation. Patients were excluded based on the following criteria: they had low-grade gliomas or their MR images did not provide clear tumor visualization due to other reasons, preventing proper annotation. Patients had a history of or concurrent brain diseases other than glioma. Patients had a history of epilepsy or the possibility of epilepsy caused by other reasons. Patients had other types of tumors, with potential for metastasis. The quality of the patients’ images was poor. The same inclusion and exclusion criteria were followed to conduct a similar search on the imaging database of Dalian Medical University. The workflow is illustrated in Figure 2.

In the end, 142 patients were included in the study, with 127 patients from Shengjing Hospital of China Medical University. Among them, 89 patients were assigned to the training set, and 38 patients were assigned to the validation set. The remaining 15 patients were from the Second Affiliated Hospital of Dalian Medical University and served as the test set.

### 2.2. ROI

**Figure 3:**
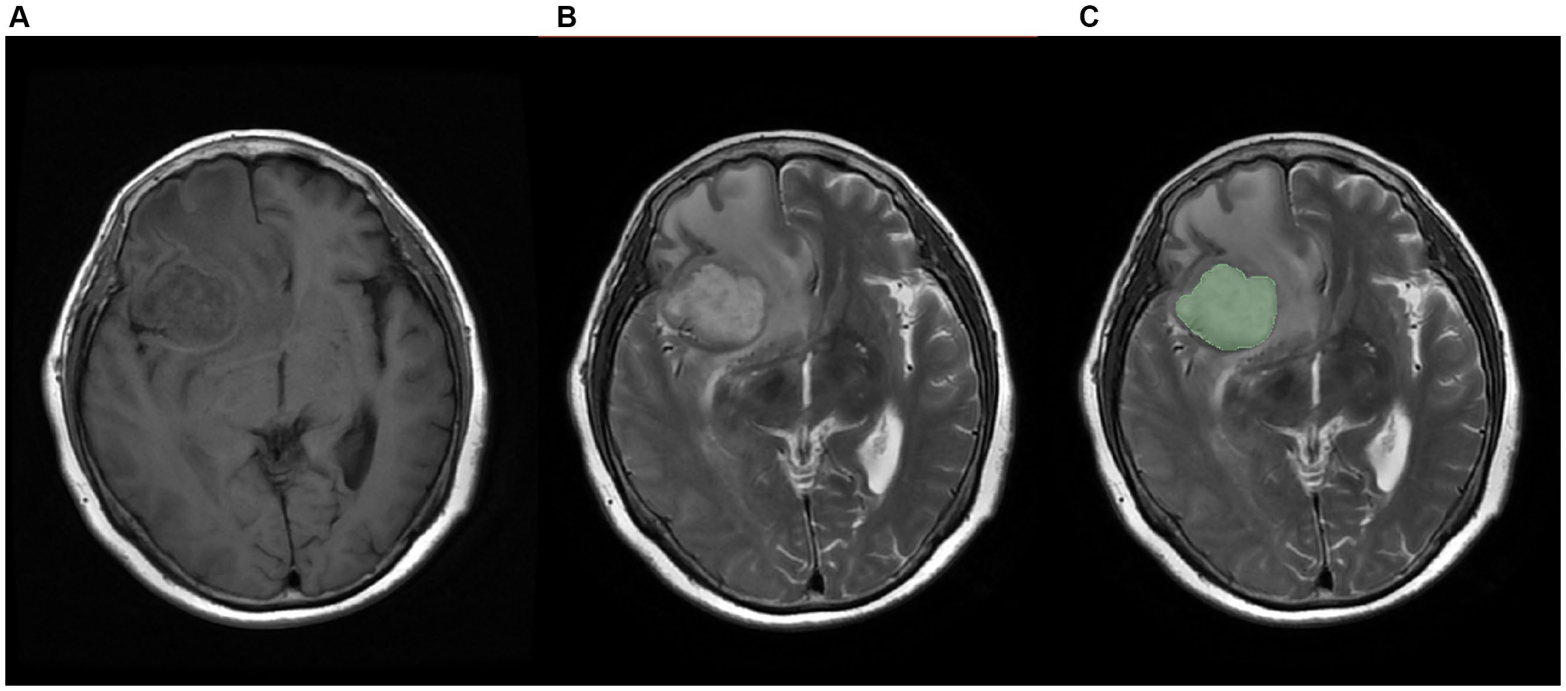
Schematic diagram of labeled images, **(A)** T1 sequence image, **(B)**T2 sequence image, **(C)** manually labeled image.

The Regions of Interest (ROI) were annotated on the T2 sequence of MRI, as shown in Figure 3. Three radiologists with more than 5 years of experience in medical imaging diagnosis used 3D-slicer (Fedorov et al., 2012) to annotate the tumor images. Ideally, the tumor did not include necrotic areas. When their opinions differed, a consensus was reached through discussion. The ROIs on the T1 sequence were obtained by image registration based on the T2 annotated ROIs.

### 2.3. Texture analysis

The Python-based pyradiomics library was used to extract radiomic features. The extracted radiomic features included 252 first-order statistics features, 14 shape-based features, 336 features based on the Gray-level co-occurrence matrix (GLCM), 224 features based on the Gray-level run-length matrix (GLRLM), 224 features based on the Gray-level size-zone matrix (GLSZM), 196 features based on the Gray-level dependence matrix (GLDM), and 70 features based on the Neighborhood gray-tone difference matrix (NGTDM).Before building the model, the radiomic features were normalized to reduce the impact of inherent differences and enhance data comparability. To reduce the number of features, the Pearson correlation coefficient (PCC) was used to compare the similarity between each feature pair. If the PCC value between two feature pairs was greater than 0.99, we randomly removed one of them to ensure that the reduced features were independent and not strongly correlated. Next, the ANOVA algorithm was applied for feature selection. The machine learning models in this study were built using the Python sklearn library. Four machine learning methods was used, namely random forests, logistic regression, support vector machines, and decision trees, to fit the data. For the patient data from Shengjing Hospital of China Medical University, the data was split into a training set and a validation set with a ratio of 7:3. The model was trained using the training set and selected the model parameters that achieved the best performance on the validation set as the final model parameters. The data from the Second Affiliated Hospital of Dalian Medical University was used as an external test set, which was not used during the training process. While accuracy was the primary metric for evaluating the model’s performance, multiple evaluation metrics were presented to assess the model comprehensively.

### 2.4. Deep learning model

**Figure 4:**
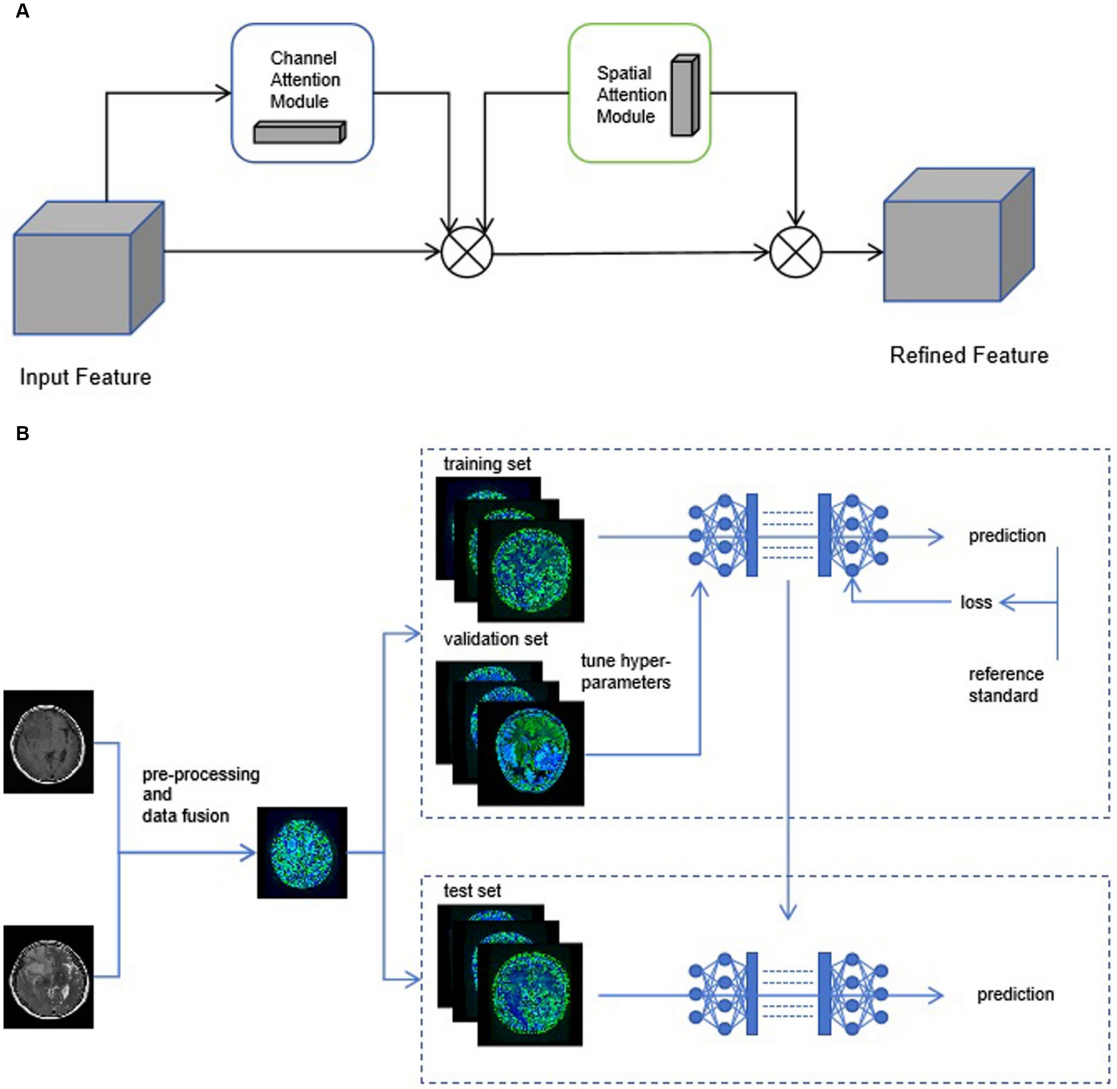
**(A)** The structure diagram of convolutional block attention module (CBAM). **(B)** We use early-fusion data to greatly improve the speed of neural network training.

Individual patient MRI cross-sections containing tumor regions were collected based on the annotations of radiologists. The patient images were standardized. The data from two sequences, T1 and T2 images, was used for training. For each patient, the T1 and T2 images of the same cross-section were fused to create a single sample. The patient data in the training, validation, and test sets were consistent with the radiomics model. Multiple CNN models were employed for patient classification, and the deep learning models were built using the Python pytorch library. AlexNet, DenseNet121, GoogleNet, MobileNet_v3_large, ResNet101, and Vgg19 were utilized as base networks, and attention mechanisms were incorporated. As shown in Figure 4, CBAM includes two independent sub-modules, the Channel Attention Module (CAM) and the Spatial Attention Module (SAM). Each network was trained for 50 epochs, and the model with the highest accuracy was saved based on the performance on the validation set. While accuracy remained the main evaluation metric, the model’s recall, F1-score, and precision were also presented to provide a comprehensive evaluation.

## 3. Results

### 3.1. Baseline characteristics of patients

The patient’s clinical information is shown in Table 1.

**Table 1:** Clinical information of the patient.

		Train	Validation	Test
**Sex**				
Male		42	17	7
Female		47	21	8
**Age**				
<44		32	14	3
45–59		57	24	12
60–74		0	0	0
75–89		0	0	0
>90		0	0	0
**Epilepsy**				
Positive		20	8	5
Negative		69	30	10

**Figure 5:**
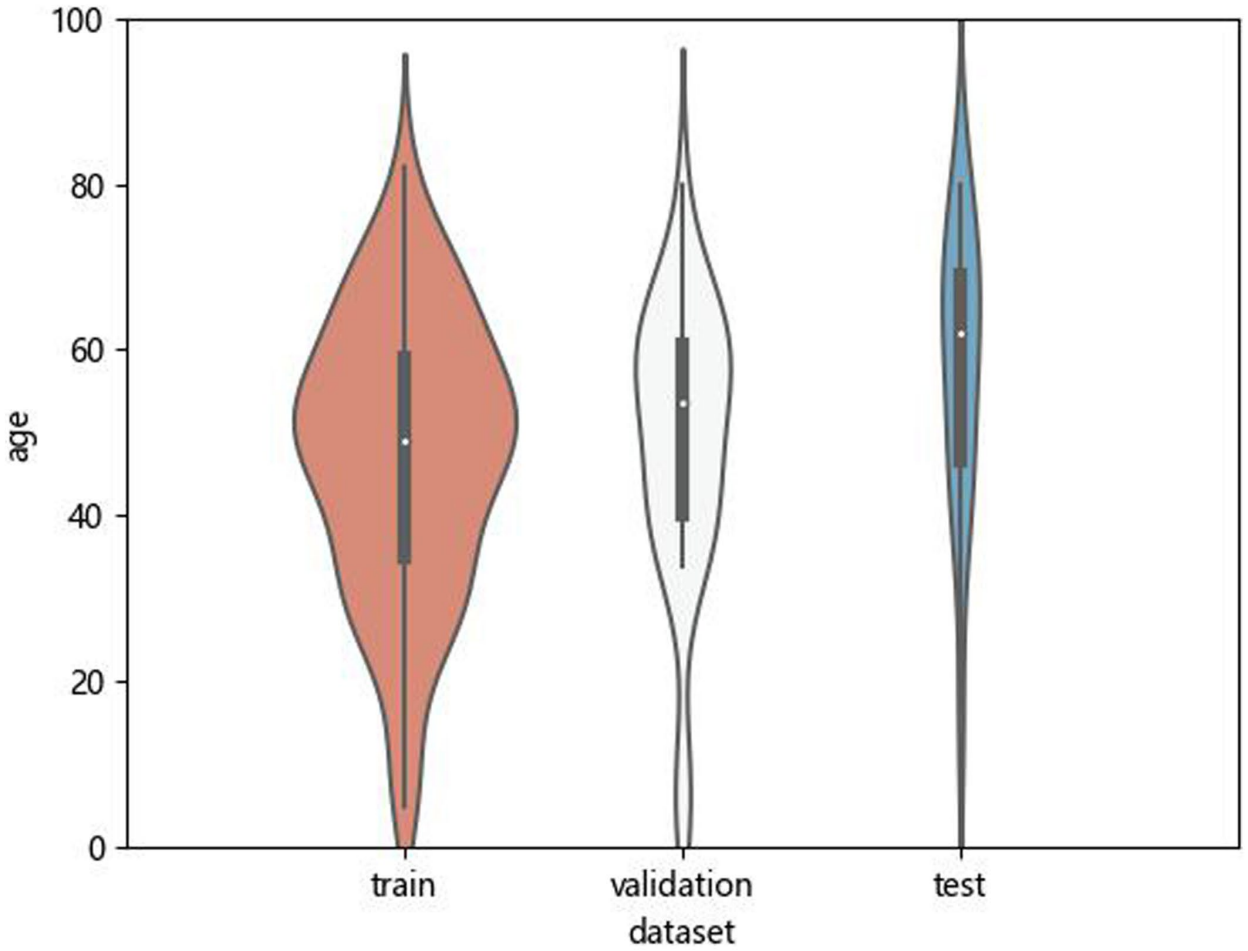
Age distribution of patients in training sets, test sets and validation sets.

The age distribution of patients in the training set, validation set and test set is shown in Figure 5. There was no significant difference in the age distribution of patients between the training set and the validation set.

**Figure 6:**
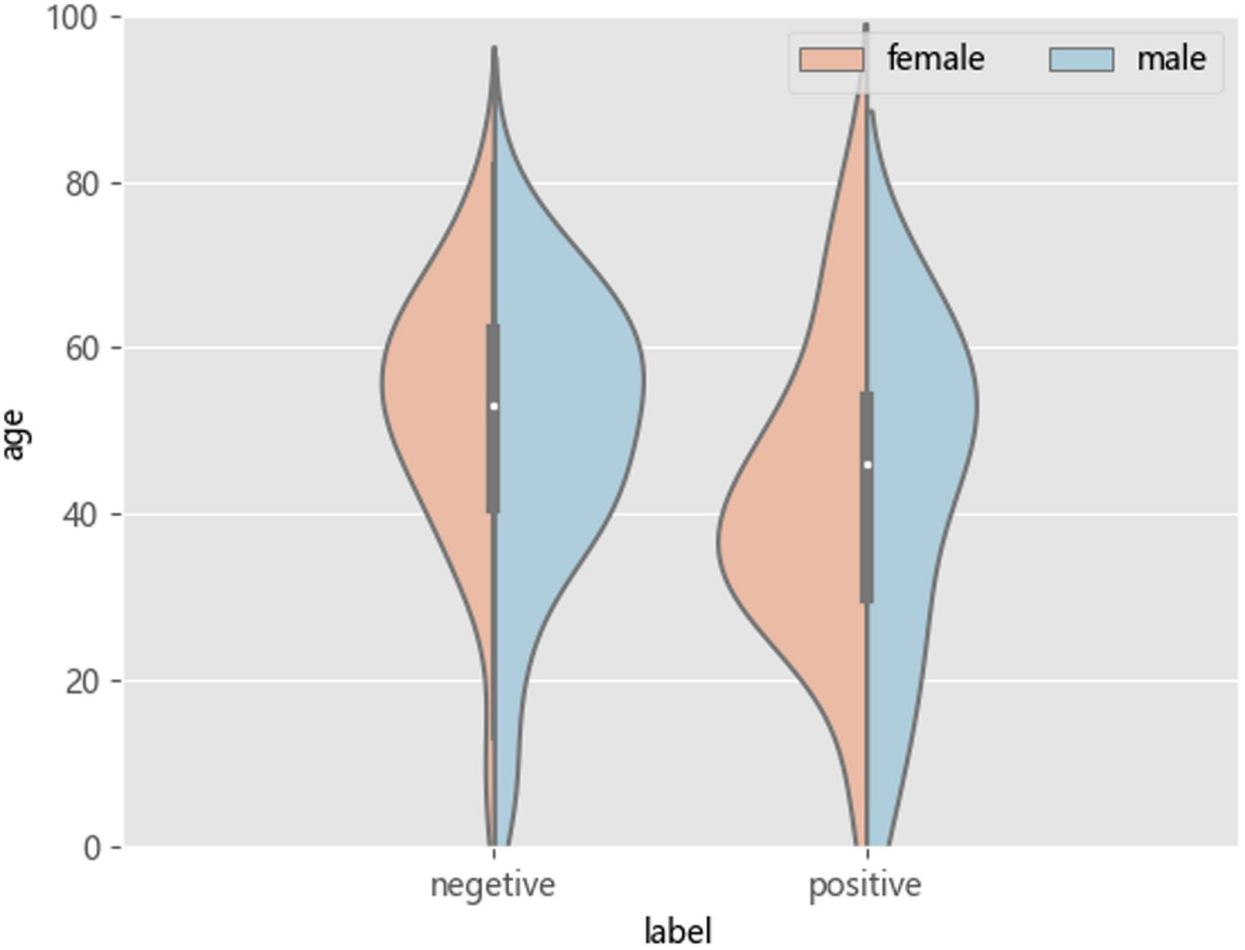
Age distribution in patients with and without epilepsy.

Among patients with and without epilepsy, the age distribution is shown in Figure 6.

### 3.2. Result of texture analysis

Considering the data distribution and preliminary experimental results, the texture parameters were individually modeled using four machine learning algorithms: Random Forest, Logistic Regression, Support Vector Machine, and Decision Tree. After feature selection, 9 texture features were included for modeling, as shown in Table 2.

**Table 2:** Selected features for machine learning algorithms.

T1_feature	T1_logarithm_gldm_DependenceVariance	T1_logarithm_glrlm_RunLengthNonUniformityNormalized	T1_original_shape_MajorAxisLength	T1_original_shape_Maximum2DDiameterColumn	T1_original_shape_Maximum2DDiameterRow	T1_original_shape_Maximum2DDiameterSlice	T1_original_shape_Maximum3DDiameter	T1_original_shape_MinorAxisLength
T2_feature	T2_original_shape_Maximum2DDiameterSlice							

**Figure 7:**
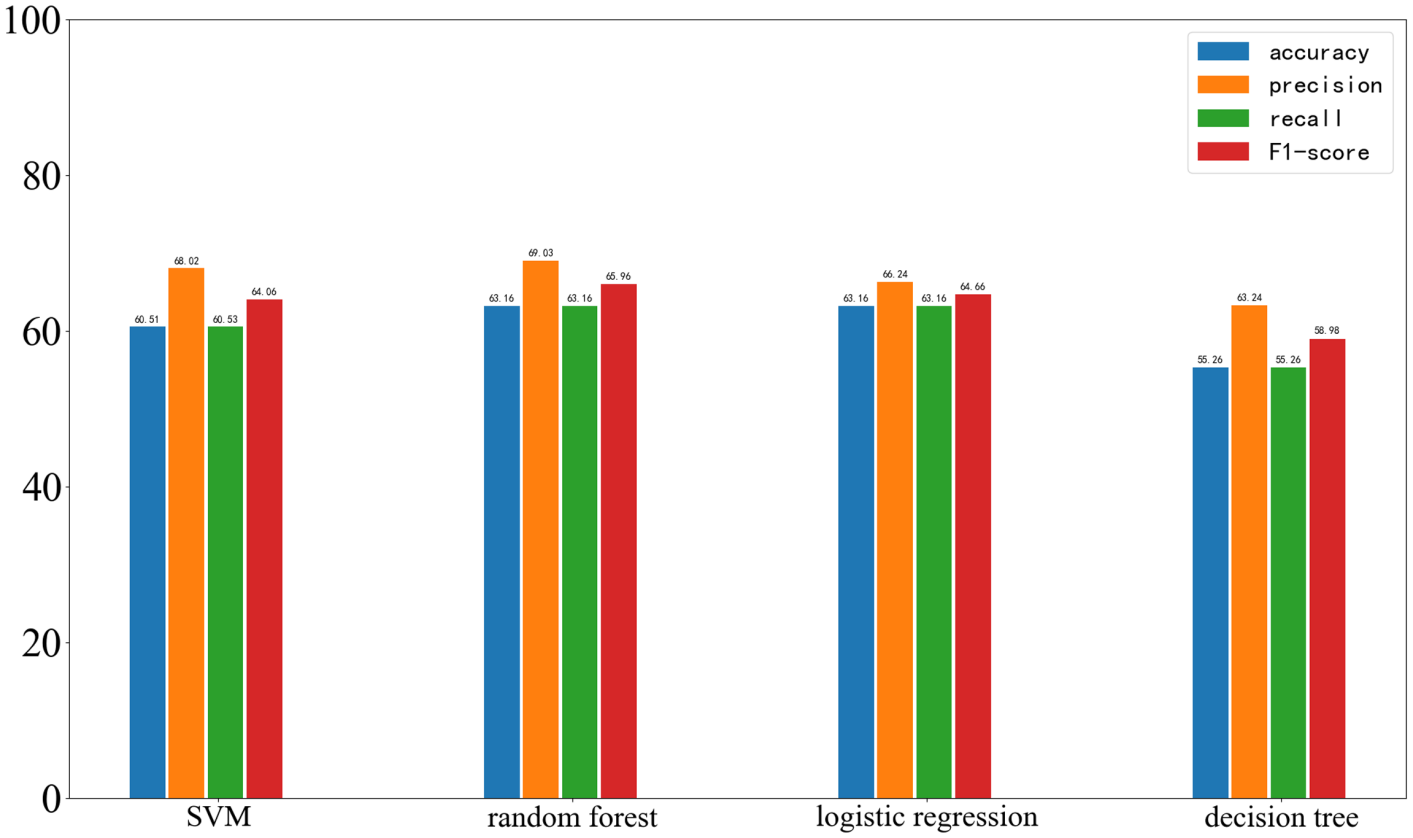
Results of Four Machine Learning Methods on the Validation Set, with the four parameters from left to right being accuracy, precision, recall, and F1-score.

The performance of the four selected algorithms on the validation set, which is from the same center as the training set, is shown in Figure 7. Among them, SVM achieved an accuracy of 60.51%, precision of 68.02%, recall of 60.53%, and F1-score of 64.06%; random forest achieved an accuracy of 63.16%, precision of 69.03%, recall of 63.16%, and F1-score of 65.96%; logistic regression achieved an accuracy of 63.16%, precision of 66.24%, recall of 63.16%, and F1-score of 64.66%; decision tree achieved an accuracy of 56.26%, precision of 63.24%, recall of 56.26%, and F1-score of 58.98%.

**Figure 8:**
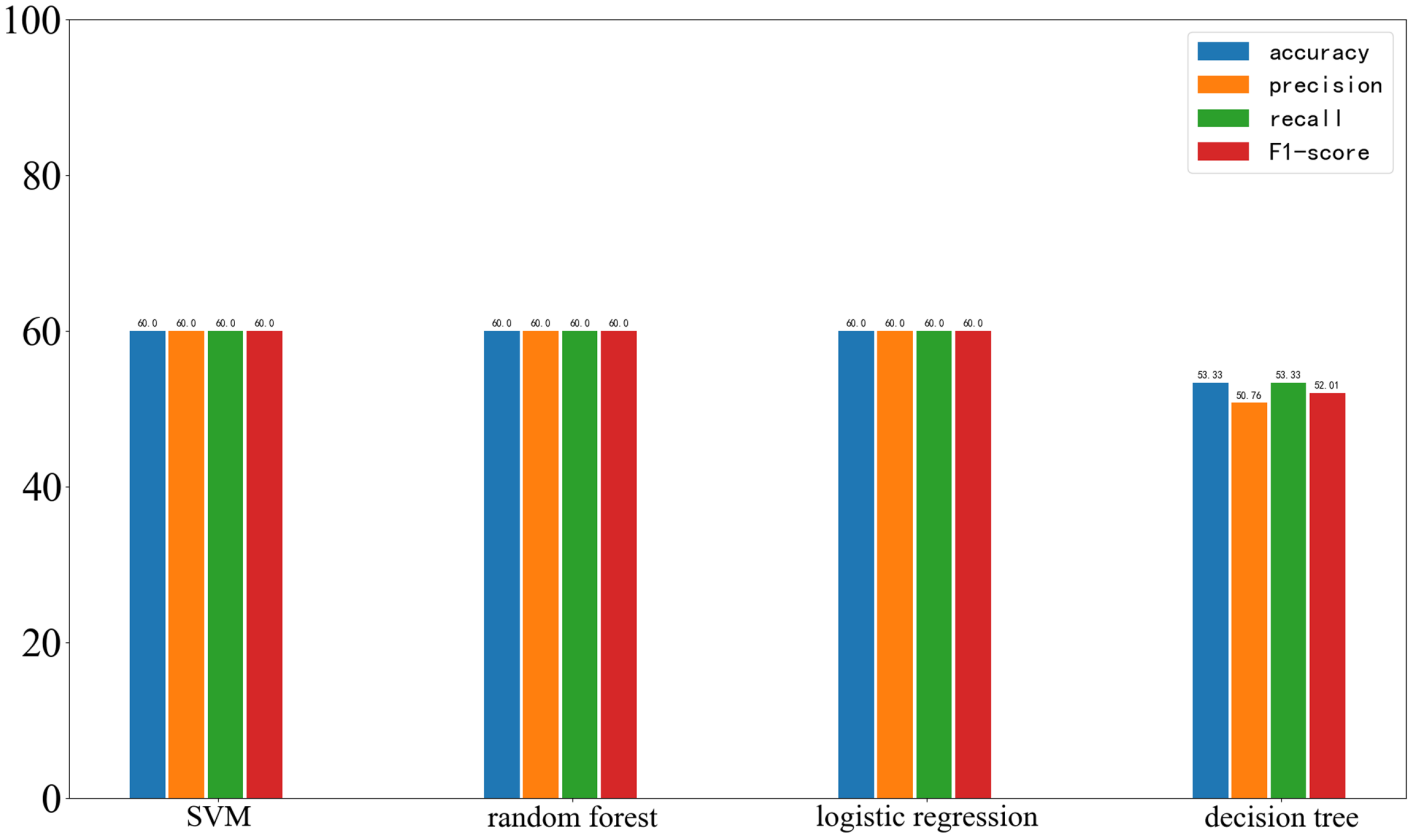
Results of the four machine learning methods on the test set, with the four parameters shown from left to right: Accuracy, Precision, Recall, and F1-score.

On the external test set from another center, the results of the four machine learning methods are as follows: SVM: accuracy 60%, precision 60%, recall 60%, F1-score 60%. Random forest: accuracy 60%, precision 60%, recall 60%, F1-score 60%. Logistic regression: accuracy 60%, precision 60%, recall 60%, F1-score 60%. Decision tree: accuracy 53.33%, precision 50.76%, recall 53.33%, F1-score 52.01%. The results of the four machine learning models are shown in Figure 8.

### 3.3. Result of deep learning model

**Figure 9:**
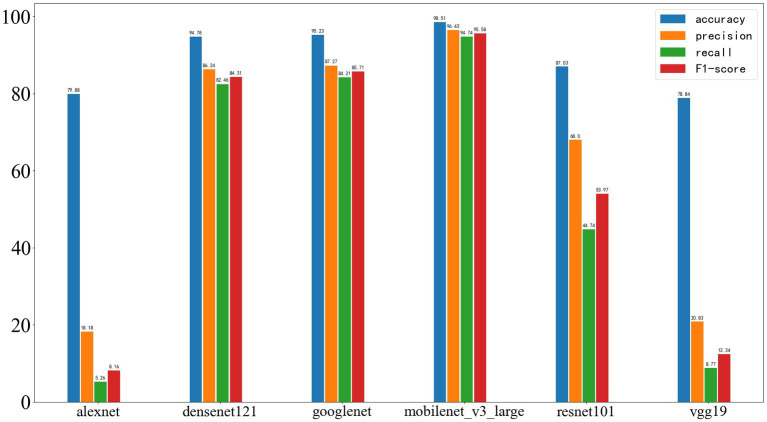
Results of deep learning on the training set, with the four performance metrics displayed from left to right: accuracy, precision, recall, and F1-score.

As shown in Figure 9, in the training dataset, all neural networks demonstrated relatively strong classification abilities.

**Figure 10:**
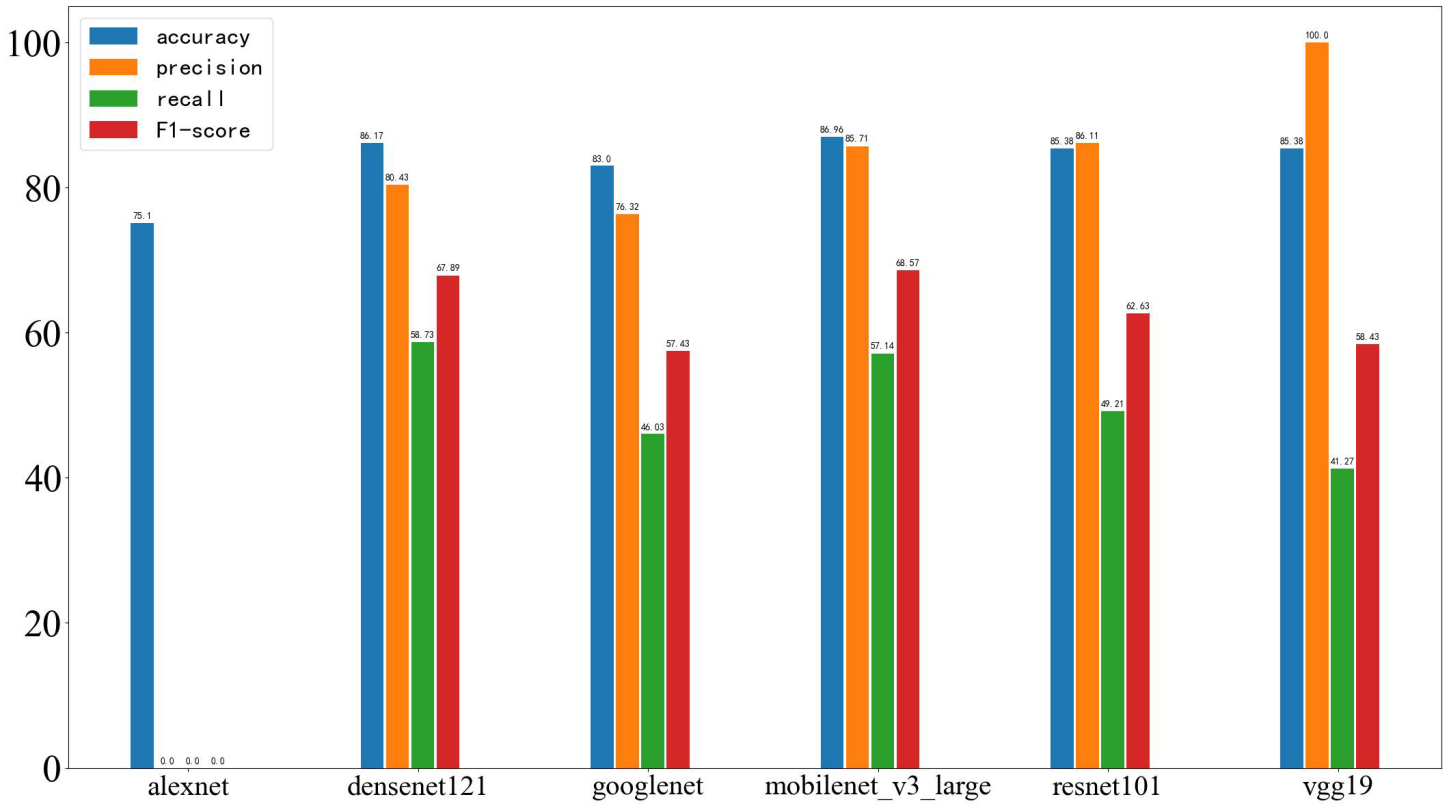
Results of deep learning on the validation set, with the four performance metrics displayed from left to right: accuracy, precision, recall, and F1-score.

On the validation set, the results were as follows: AlexNet achieved an accuracy of 75.1%, precision of 0, recall of 0, and F1-score of 0; DenseNet121 achieved an accuracy of 86.17%, precision of 80.43%, recall of 58.73%, and F1-score of 67.89%; GoogleNet achieved an accuracy of 83.00%, precision of 76.32%, recall of 46.03%, and F1-score of 57.43%; MobileNet_v3 achieved an accuracy of 86.96%, precision of 85.71%, recall of 57.14%, and F1-score of 68.57%; ResNet101 achieved an accuracy of 85.38%, precision of 86.11%, recall of 49.21%, and F1-score of 62.63%; VGG19 achieved an accuracy of 85.38%, precision of 100%, recall of 41.27%, and F1-score of 58.43%. The results are shown in Figure 10.

**Figure 11:**
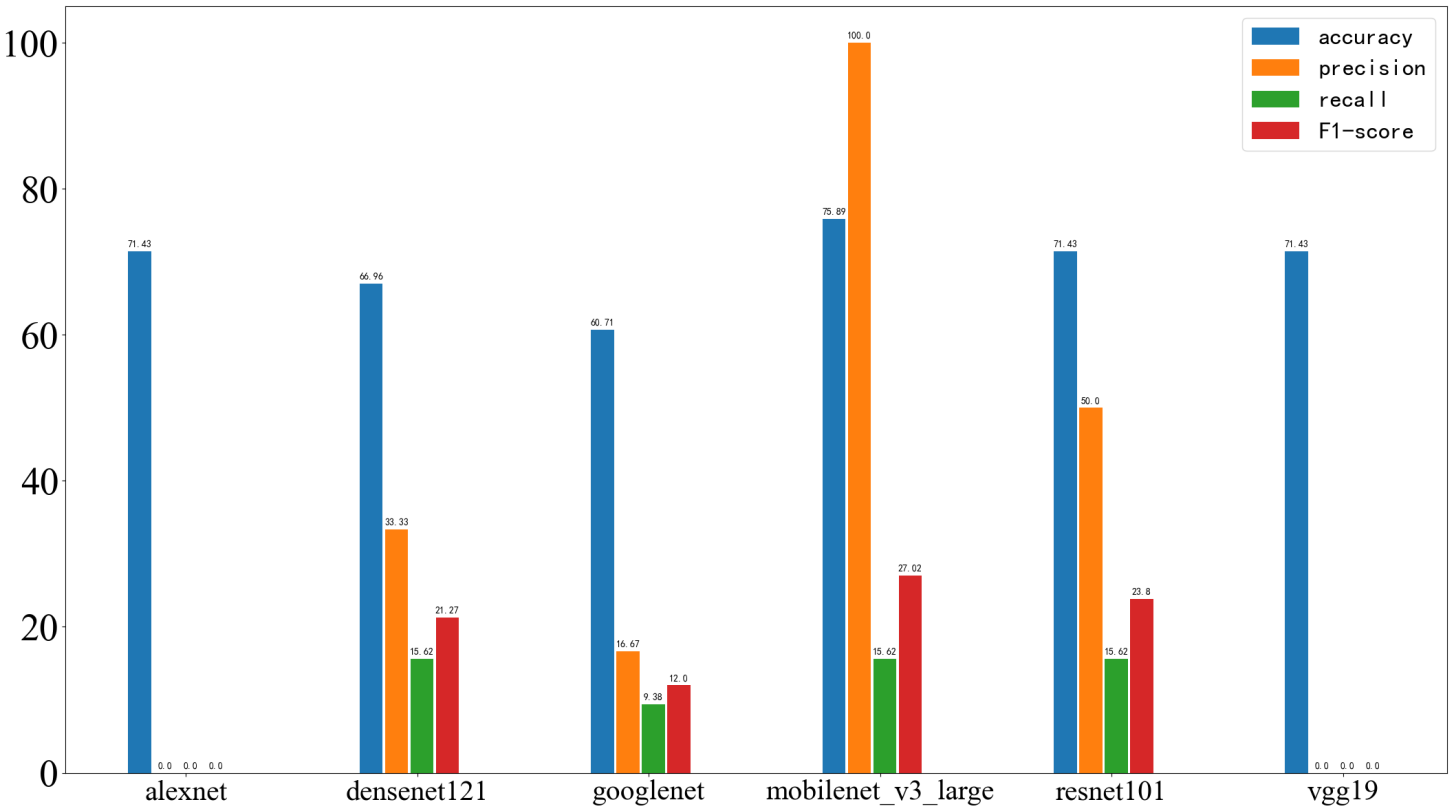
Deep learning results on the test set, with the four metrics displayed from left to right as accuracy, precision, recall, and F1-score.

Figure 11 shows the results on the external test set. The neural networks based on AlexNet and VGG19 did not demonstrate acceptable classification performance. The models based on DenseNet121 and GoogleNet experienced a significant drop in classification ability, while those based on MobileNet_v3 and ResNet101 also showed a certain decrease in performance. On the test set, the accuracy of AlexNet was 71.43%, precision was 0, recall was 0, and F1-score was 0; DenseNet121 achieved an accuracy of 66.96%, precision of 33.33%, recall of 15.62%, and F1-score of 21.27%; GoogleNet achieved an accuracy of 60.71%, precision of 16.67%, recall of 9.38%, and F1-score of 12.00%; MobileNet_v3 achieved an accuracy of 75.89%, precision of 100.00%, recall of 15.62%, and F1-score of 27.02%; ResNet101 achieved an accuracy of 71.43%, precision of 50.00%, recall of 15.62%, and F1-score of 23.80%; VGG19 achieved an accuracy of 71.43%, precision of 0, recall of 0, and F1-score of 0.

**Figure 12:**
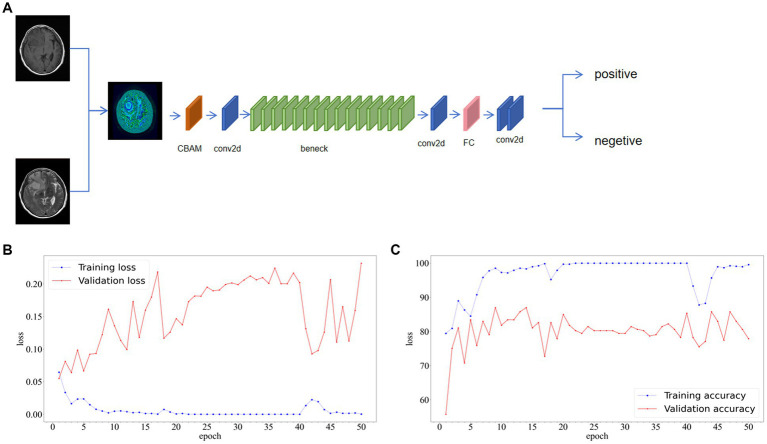
**(A)** Structure diagram, **(B)** Loss curve and **(C)** accuracy curve of GE-Net.

In this study, the best-performing network has been named as GE-Net, and its structure and the loss curve and accuracy curve of the GE-Net during 50 epochs of training are shown in Figure 12. Meanwhile, based on the predictions for each patient in the test dataset, we adopted the majority vote strategy to determine the overall prediction for that patient. Under this approach, GE-Net achieved an accuracy of 73.3%. Results of machine learning and deep learning models are shown in Table 3. We constructed a dataset using MRIs from 10 patients with meningiomas from Shengjing Hospital, affiliated with China Medical University. Among them, 5 patients exhibited epileptic symptoms, while the other 5 did not. We applied the same process to process the images of these patients and tested GE-Net, resulting in an accuracy of 62.22%.

**Table 3:** Results of machine learning and deep learning models.

	Accuracy	Precision	Recall	F1-score
SVM	60.00%	60.00%	60.00%	60.00%
Random forest	60.00%	60.00%	60.00%	60.00%
Logistic regression	60.00%	60.00%	60.00%	60.00%
Decision tree	53.33%	50.76%	53.33%	52.01%
AlexNet	71.43%	0.00%	0.00%	0.00%
DenseNet121	66.96%	33.33%	15.62%	21.27%
GoogleNet	60.71%	16.67%	9.38%	12.00%
MobileNet_v3_large	75.89%	100.00%	15.62%	27.02%
ResNet101	71.43%	50.00%	15.62%	23.80%
Vgg19	71.43%	0.00%	0.00%	0.00%

## 4. Discussion

As awareness of the right to health has increased, becoming infected with non-communicable diseases is now viewed by society as an infringement upon this right. Injury identification by forensic doctors is an important process in judicial practice. Both parties to the dispute will negotiate the amount of compensation based on the results of the injury evaluation. According to different injury types, the difficulty of forensic identification of injury varies greatly. For organic injuries, including trauma, the cause of the injury is clear and therefore can be evaluated more easily. In the identification of injuries associated with pathogen infection, including human cytomegalovirus, forensic doctors should not only consider the immediate damage caused by infection, but also consider the damage caused by tumors directly related to viral infection and other diseases caused by tumors. In the absence of quantitative criteria and objective indicators, the identification of such injuries undoubtedly poses a huge challenge for forensic medicine. Human cytomegalovirus, as a virus that can be transmitted through re-exposure, has been demonstrated to cause various harms to infected individuals. Glioma, one of the most prognosis-poor malignant brain tumors, has been linked to cytomegalovirus. However, in forensic practice, identifying the damage suffered by patients due to glioma-related to cytomegalovirus remains challenging. This is primarily because epilepsy is a crucial determinant in injury assessment, and glioma-related epilepsy presents significant uncertainty. Evaluating the possibility of glioma-related epilepsy in infected individuals without disrupting their treatment is of paramount importance for accurate assessments.

In this study, a total of 142 patients diagnosed with glioma from two centers were included. We determined whether patients had exhibited typical epilepsy symptoms based on their clinical presentations. In this research, preoperative MR images of patients, specifically T1 and T2 sequences, were used to construct a deep learning predictive model. To demonstrate the advantages of the model proposed in this study, a radiomic model was employed for comparison. The model presented in this study, which is based on MobileNetV3 with added attention mechanisms, exhibited significant superiority, a superiority observed across the training set, validation set, and test set.

In this study, T1 and T2 sequences were included to establish machine learning models. Compared to machine learning, neural network models exhibited a significant advantage on the test set. However, on the external test set, the classification performance of the machine learning model decreased. Several factors might contribute to this phenomenon. Firstly, it could be attributed to inherent differences in MR images from different centers, making machine learning models reliant on a limited number of parameters less robust. Secondly, the final machine learning model was essentially constructed using only a subset of texture features. During feature extraction, thousands of texture features were generated, and although the validation set was used for model selection, it was challenging to fully address issues of model overfitting. Finally, the limited size of the test set might not adequately reflect the model’s classification capability, thereby impacting the machine learning model’s performance on external data. This highlights the importance of considering the differences in data from various centers, which has often been overlooked in previous research where single-center data was commonly used for modeling [Sun et al., 2020; Wang et al., 2020; Gao et al., 2021; National Guideline Centre (UK), 2022]. It is worth noting that this decline in classification performance on the training set was also observed in the MobileNet_v3 model, which yielded the best results. This model’s performance on the test set still lagged behind that on the training and validation sets, which underscores the impact of inter-device differences resulting in image disparities. We tested GE-Net using data from patients with meningiomas, and the accuracy showed a significant difference compared to the test set composed of glioma patients. This further confirms that the occurrence of epilepsy in glioma patients depends not only on the extent of tumor invasion and its growth location but is also highly correlated with the type and growth characteristics of the tumor. In comparison to studies that only included a single sequence, this research incorporated a more comprehensive set of medical information related to epilepsy caused by brain tumors, thus enhancing the model’s accuracy. This improvement can be attributed to the unique characteristics of the T1-weighted sequence, where substances with short T1 relaxation times, such as fat, melanin, and protein, exhibit bright high signals, while cerebrospinal fluid (CSF) appears as a low signal, making it suitable for anatomical observation. The T2 sequence, on the other hand, provides a relatively better representation of tumor and lesion states. It is generally accepted that epilepsy caused by brain tumors involves various factors, including the tumor itself and its location. Therefore, the inclusion of data from both T1 and T2 sequences is advantageous for enhancing the model’s effectiveness. In addition to MRI, CT scans and EEG (Electroencephalogram) are commonly used diagnostic tests for epilepsy patients. CT scans can provide clear visualization of brain structures, making them particularly advantageous for detecting organic lesions like calcified brain injuries. However, CT images typically have lower resolution and may not effectively identify around 50% of the structural abnormalities that cause seizures. Especially in the case of temporal lobe epilepsy, CT’s ability to identify lesions is even poorer, and it may not provide sufficient information for neural network models (Shaikh et al., 2019). Currently, EEG remains a crucial test for epilepsy diagnosis with high specificity. Generally, sensitivity can also be significantly improved through repeated measurements. However, EEG is influenced by various factors and may have difficulty capturing abnormal discharges during seizure-free intervals, which can, in fact, increase the challenge of obtaining effective training data for artificial intelligence models (Benbadis et al., 2020).

In this study, approximately 23.24% of patients experienced seizures, and a significant proportion of them suffered primarily from physical injuries during seizure episodes. Nearly all epilepsy patients underwent drug therapy to control their seizure symptoms. Regardless of whether seizures occurred, all patients underwent surgery within the first week of hospitalization. These statistics align with the seizure incidence observed in some previous research but differ significantly from the seizure rates reported in other studies among glioma patients. However, this underscores the potential of this study in forensic assessments. The substantial variability in these statistical data may be related to the timing of surgery, indicating that a significant portion of patients experienced a resolution of their epilepsy symptoms due to early surgical intervention. Additionally, in clinical practice, the use of antiepileptic drugs remains the preferred choice for healthcare providers. This treatment approach is widely accepted by patients, possibly because symptom control after receiving medication provides them with psychological comfort (Siomin et al., 2005). However, the harm inflicted on patients by gliomas has not diminished as a result. One study indicated that glioma patients receiving levetiracetam for prevention or treatment, despite robust control of seizure symptoms, did not experience significant benefits (Boison, 2010). This implies that regardless of the method used to control or eliminate seizures, the harm caused by the tumor to the patient is underestimated. Therefore, this study provides robust support for forensic assessments of the damage caused by cytomegalovirus reinfection through the prediction of whether patients will experience seizures based on preoperative MR scans.

Nevertheless, this study has certain limitations. Firstly, the diagnosis of epilepsy in enrolled patients was primarily based on clinical presentation, and thus, it cannot entirely rule out the possibility of seizures due to other causes. Secondly, this study utilized deep learning to predict whether glioma patients would develop epilepsy. Limited by the nature of deep learning, the ability to interpret the features extracted by the model is restricted. Thirdly, due to the difficulty in obtaining patients who never underwent surgical treatment or took antiepileptic drugs until clinical outcomes were reached, this research could not encompass all scenarios of glioma-related epilepsy. Lastly, the model is derived from only two centers, and the data of epileptic and non-epileptic patients is not perfectly balanced.

## 5. Conclusion

In this study, a deep learning model was developed based on head MRIs to predict whether glioma patients would develop epilepsy. The data was incorporated from two different centers to validate the model’s robustness, and through comparisons with other artificial intelligence methods, which demonstrated the superiority of model. This research can assist forensic experts in more accurately identifying the damage caused by glioma associated with cytomegalovirus infection, thereby providing support in cases involving infringements on the right to health.

## Data availability statement

The raw data supporting the conclusions of this article will be made available by the authors, without undue reservation.

## Ethics statement

The studies involving humans were approved by Ethics Committee of the Second Affiliated Hospital of Dalian Medical University and Medical Ethics Committee of Shengjing Hospital Affiliated to China Medical University. The studies were conducted in accordance with the local legislation and institutional requirements. Written informed consent for participation was not required from the participants or the participants’ legal guardians/next of kin in accordance with the national legislation and institutional requirements.

## Author contributions

WW: Data curation, Formal analysis, Investigation, Methodology, Writing – original draft. XL: Software, Validation, Writing – original draft. LY: Visualization, Writing – original draft. JY: Funding acquisition, Supervision, Writing – review & editing.
